# An Integrated IoT-Based Multi-Sensor Framework for Real-Time Indoor Environment and Safety Monitoring

**DOI:** 10.3390/s26123702

**Published:** 2026-06-10

**Authors:** Aung Min Naing, Duaa Zuhair Al-Hamid, Anuradha Singh

**Affiliations:** 1 Department of Computer and Information Sciences, Auckland University of Technology (AUT), Auckland 1010, New Zealand; qcv9766@autuni.ac.nz (A.M.N.); duaa.alhamid@aut.ac.nz (D.Z.A.-H.); 2 Department of Data Science and Artificial Intelligence, Auckland University of Technology (AUT), Auckland 1010, New Zealand

**Keywords:** IoT, indoor environment monitoring, multi-sensor integration, real-time analytics, MQTT protocol, edge computing, smart cities, indoor air quality

## Abstract

Poor indoor air quality, inadequate ventilation, and unnoticed local disturbances can reduce occupant well-being and compromise practical safety in smart-home and small-building environments. Although low-cost Internet-of-Things (IoT) sensing technologies are widely available, many monitoring systems remain focused on single-modality sensing and do not jointly evaluate environmental conditions, vibration activity, communication reliability, and gateway-side interpretation within one framework. This study presents the design, implementation, and proof-of-concept evaluation of a low-cost, privacy-conscious, non-imaging IoT-based indoor environment and safety-awareness monitoring framework built with ESP32/Arduino sensor nodes and a Raspberry Pi gateway. The system integrates carbon dioxide, temperature, humidity, gas-resistance/VOC-trend indication, and vibration sensing with MQTT-based communication and edge-side analytics. Controlled subsystem experiments showed that CO_2_ concentration differentiated ventilation conditions, increasing from 395.47 ppm in the valid empty/open-door baseline to 1083.16 ppm in the closed occupied condition. Vibration states were distinguished using root-mean-square acceleration features across calm, surface-disturbance, footstep, play, and jump conditions. MQTT evaluation using 1000-message batches showed no observed message loss or duplicates across the tested QoS/network combinations, although latency and throughput varied by network configuration and QoS level. QoS 1 provided a practical balance between low latency and protocol-level delivery assurance in the tested local/Wi-Fi setting. A final integrated validation run further demonstrated synchronized acquisition from indoor environmental, vibration, and outdoor CO_2_ reference publishers through the same Raspberry Pi gateway, with zero missing or duplicate sequence flags across the three streams. Overall, the findings indicate that lightweight open-source IoT hardware can support a reproducible building-level sensing and edge-analytics prototype for indoor environment and safety-awareness monitoring. Broader deployment in standard-sized rooms, multi-room buildings, and smart-city infrastructure remains future work.

## 1. Introduction

Smart cities increasingly rely on distributed IoT infrastructure to monitor environmental quality, building comfort, and safety across residential and commercial spaces. In this context, smart homes and small offices can be viewed as building-level sensing endpoints that generate localized information about ventilation, environmental comfort, and local disturbance events. Indoor environments remain important because poor ventilation and air-quality degradation are commonly interpreted through indicators such as carbon dioxide (CO_2_), humidity, and gas-resistance/VOC-trend patterns. CO_2_ is not a complete measure of indoor air quality, but it is a practical indicator of ventilation adequacy and human bioeffluent accumulation [1,2]. Controlled chamber and building-environment studies have reported measurable associations between elevated CO_2_, ventilation conditions, VOC exposure, and cognitive-performance outcomes, especially for complex decision-making tasks [1,3,4]. However, the literature also indicates that effects vary by exposure design and task type; therefore, CO_2_ should be framed as a ventilation and risk-awareness indicator rather than as a medical diagnostic variable [5].

Recent IoT research shows that compact environmental sensing systems can monitor CO_2_ and other indoor parameters effectively in domestic and building settings. ESP32-based and off-the-shelf IAQ systems have demonstrated continuous monitoring, dashboard visualization, and near-real-time data access [6,7,8,9]. Multi-node IAQ systems have also shown the practicality of using Sensirion SCD41 CO_2_ sensors in open-source wireless indoor monitoring deployments [10]. At the same time, smart-building research has emphasized privacy-aware sensing for occupancy interpretation and environmental control, especially where camera-based systems are undesirable [11,12,13,14]. Nevertheless, many systems remain functionally narrow: environmental monitoring is often studied independently from local disturbance sensing, while communication reliability is assumed rather than experimentally measured.

This limitation matters because a home-monitoring system is only useful if it can both detect meaningful indoor-state changes and deliver that information reliably in real time. Vibration-based monitoring has long been used in industrial condition monitoring and structural-health applications to identify imbalance, impact, wear, and abnormal dynamic behavior [15,16,17]. Low-cost MEMS accelerometers can provide practical alternatives to expensive commercial vibration sensors when the goal is distributed or resource-constrained condition awareness rather than certification-grade structural diagnosis [18,19]. Similarly, MQTT is widely used in IoT systems because of its lightweight publish/subscribe model, low overhead, and configurable quality-of-service levels [20,21,22,23,24,25,26]. Despite this, limited experimental work combines environmental sensing, vibration sensing, and protocol-level communication evaluation into one low-cost, edge-enabled smart-home framework that can also be interpreted as a small building-level component of future smart-building IoT infrastructure.

This study addresses that gap by designing and evaluating an IoT-based home environment and safety monitoring system that integrates environmental sensing, vibration sensing, and MQTT communication-performance analysis within one edge-enabled architecture. The work focuses on proof-of-concept validation in a compact indoor setting using open-source hardware and privacy-conscious, non-imaging sensors. The aim is to validate a reproducible building-level sensing and edge-analytics component that demonstrates integration feasibility and could later be scaled toward multi-room, small-office, assisted-living, or smart-building deployments after further validation.

The main contributions of this paper are as follows:

- We propose a low-cost, open-source IoT architecture that integrates indoor environmental sensing, vibration sensing, and gateway-based edge analytics within a single monitoring framework.
- We propose a low-cost, open-source edge-IoT monitoring framework that integrates indoor environmental sensing, vibration-based local-disturbance detection, MQTT communication, gateway-side synchronization, local dashboard visualization, and edge analytics within one unified building-level pipeline.
- We develop and validate the framework through a two-stage design pathway, progressing from a serial feasibility prototype to a distributed MQTT-based implementation with multiple sensing publishers and a Raspberry Pi edge gateway.
- We demonstrate that the integrated sensing pipeline can capture complementary indoor-state evidence, where environmental measurements reflect gradual ventilation-related changes and triaxial vibration features capture short-duration disturbance events.
- We evaluate MQTT QoS behavior within the operating monitoring framework, showing how latency, throughput, and protocol-level delivery assurance affect practical gateway-side interpretation under localhost and Wi-Fi settings.
- We conduct an integrated end-to-end validation run showing synchronized multi-node MQTT acquisition, dashboard monitoring, local CSV logging, message-health assessment, and gateway-side fusion-state analysis within one edge-processing workflow.

Beyond the main contribution list, the resulting reference model may inform future smart-home, small-office, assisted-living, and smart-building monitoring studies after further validation in larger and more diverse deployment settings.

## 2. Related Work

### 2.1. IoT-Based Indoor Air-Quality Monitoring

Indoor air-quality monitoring is a major topic in smart-building and environmental-health research because people spend most of their time indoors and are therefore highly exposed to indoor-generated pollutants. Among commonly monitored variables, CO_2_ is widely used as an indicator of ventilation adequacy and occupancy-related air-quality degradation [1,2]. Satish et al. exposed participants to 600, 1000, and 2500 ppm CO_2_ in a controlled office-like chamber and reported statistically significant reductions in several decision-making performance scales at 1000 ppm and stronger reductions at 2500 ppm [1]. Allen et al. further showed that office workers’ cognitive scores were associated with CO_2_, ventilation, and VOC exposure under controlled office-environment conditions [3]. Snow et al. examined short-duration exposure to elevated CO_2_ using physiological and EEG-based measurements and reported evidence consistent with possible effects on cognitive-performance patterns, while also calling for further work [4]. Additional intervention-oriented work has explored whether negative air ions can influence cognitive and health outcomes under high-purity CO_2_ exposure, but such mitigation strategies remain outside the scope of the present design and are cited only to contextualize the broader CO_2_-exposure literature [27].

Recent IoT-based systems show that low-cost embedded hardware can support continuous environmental sensing while enabling remote visualization and data access. Mota et al. developed an ESP32-based IAQ architecture that measured CO_2_ and particulate matter and transmitted data via MQTT to a remote database [7]. Their sleep-period case study showed that a slightly open bedroom door led to slower CO_2_ accumulation and lower peak concentrations than a closed-door scenario. In a related study, Mota et al. demonstrated a Matter-enabled CO_2_ sensor integrated into a smart-home ecosystem, highlighting interoperability and user-facing deployment [6]. Ramadan et al. extended indoor environmental monitoring toward institutional healthcare settings by combining multi-parameter IAQ sensing with federated learning for HVAC optimization [28].

Additional studies strengthen the technical basis for the present system. Gül and Eroğlu classified CO_2_ sensor technologies for IoT-enabled IAQ monitoring and compared NDIR, MOS/MOX, capacitive, photoacoustic, and electrochemical approaches across accuracy, response time, sensitivity, cost, durability, and application suitability [29]. Hippe et al. demonstrated an open-source wireless IAQ monitor using the Sensirion SCD41 CO_2_ sensor with PM_2.5_ and airflow sensing in a multi-node system [10]. Yasin et al. presented an off-the-shelf IAQ framework covering sensing devices, gateways, cloud platforms, data management, and dashboards [9]. Tsang et al. further demonstrated the value of long-term building-scale IEQ monitoring by deploying an IoT-based wireless sensing network in a Hong Kong office skyscraper for 15 months, collecting 1 min data from 12 locations across temperature, radiant temperature, relative humidity, air velocity, CO_2_, particulate matter, illuminance, and sound-pressure measurements [30].

For VOC-related sensing, the BME680 is useful as a compact temperature, humidity, pressure, and gas-resistance sensor, but its MOX gas response must be interpreted carefully. Palacín et al. used an array of BME680 MOX sensors for volatile classification and highlighted the cross-sensitivity and non-specificity of low-cost MOX gas sensors [31]. Consequently, the BME680 is treated in this study as an auxiliary VOC/IAQ trend and gas-resistance indicator, not as a selective gas-identification instrument.

### 2.2. Privacy-Aware Occupancy Detection in Smart Environments

Occupancy detection is important in smart environments because occupancy information improves ventilation control, energy management, and contextual interpretation of indoor conditions. Chaudhari et al. reviewed occupancy detection methods for smart buildings and showed that modern approaches range from motion and acoustic sensing to camera-based and HVAC-related sensing [11]. Their review also emphasized privacy, sensor placement, and real-world deployment constraints.

More privacy-conscious work has focused on ambient and environmental sensing rather than imaging. Fährmann et al. proposed a privacy-conscious multi-occupant detection method using ambient time-series data and reported strong classification performance in smart environments [12]. Wilhelm et al. examined human presence detection through indoor CO_2_ monitoring and argued that CO_2_ can reveal presence or absence without the viewing-angle limitations of motion sensors or the privacy concerns of cameras [13]. Gao et al. further showed that occupant behavior strongly influences building-control outcomes, reinforcing the interpretation of indoor sensing systems as human–environment systems rather than purely technical infrastructures [14].

For the present study, occupancy inference is not the primary objective. Instead, this literature supports the privacy-conscious design philosophy and justifies the use of environmental signals as indirect indicators of room state and ventilation adequacy.

### 2.3. Vibration and Structural-Condition Monitoring

Vibration-based monitoring is widely used in predictive maintenance, structural-health monitoring, and condition assessment because vibration signatures often contain early indicators of imbalance, wear, impact, or damage. Hassan et al. reviewed vibration sensors for condition monitoring and highlighted the importance of accelerometer selection and data-collection strategy in real-world deployments [15]. Lei and Wu proposed a wireless vibration monitoring system for rotating machinery and combined embedded sensing with sparse-Bayesian signal reconstruction, demonstrating the feasibility of distributed wireless vibration-state monitoring [16]. Muttillo et al. designed an IoT-based structural-health monitoring platform using triaxial accelerometer nodes and showed that their estimated damage indicator increased when the test structure was perturbed [17].

Low-cost sensor literature further supports MEMS-based vibration monitoring. Komarizadehasl et al. developed an Arduino-based low-cost structural-vibration measurement system using multiple accelerometers and reported improved low-frequency, low-amplitude measurement compared with higher-cost alternatives [18]. Komary et al. reviewed low-cost sensors for structural assessment and emphasized that affordable sensing can increase the number of measurement points available for monitoring [19]. De Simone et al. proposed an IoT-based predictive-maintenance and structural-health monitoring system using a Raspberry Pi and a low-cost MEMS accelerometer, linking vibration monitoring with visualization and future data-driven prediction [32].

Most vibration studies remain industrial, structural, or mechanical-condition oriented. They are rarely integrated with environmental sensing in domestic IoT systems. This leaves a useful research space for household-scale architecture in which vibration sensing operates as a complementary local-disturbance modality rather than as a standalone structural-diagnosis subsystem.

### 2.4. MQTT Communication Reliability in IoT Systems

MQTT is widely adopted in IoT systems because of its lightweight publish/subscribe model and suitability for constrained devices. Lee et al. analyzed MQTT loss and delay under different QoS levels and showed that higher QoS improves delivery assurance at the cost of higher end-to-end delay [20]. In MQTT, QoS 0 sends a message without acknowledgment, QoS 1 provides at-least-once delivery with possible duplicates, and QoS 2 uses a multi-step handshake to provide the strongest delivery semantics at the cost of additional overhead [20].

Chen and Kunz compared MQTT with CoAP, DDS, and a custom UDP protocol under constrained wireless conditions and showed that protocol choice has measurable consequences for latency, bandwidth, and reliability [21]. Ahlawat et al. studied MQTT over TCP within a ground-station-centric IoT framework and highlighted its relevance for scalable monitored communication [22]. Additional MQTT studies support the protocol choice and its practical limitations. Atmoko et al. described MQTT’s brokered publish/subscribe structure, QoS levels, Mosquitto broker use, and web/mobile data access for real-time sensor acquisition [23]. Hwang et al. argued that although MQTT provides delivery assurance through QoS, message ordering and missing-message recovery may require sequence tracking and application-level reliability mechanisms [24]. Quincozes et al. reviewed MQTT, MQTT-SN, CoAP, and HTTP, emphasizing IoT requirements such as security, interoperability, energy efficiency, and experimentation tools [25]. Maroșan et al. demonstrated ESP32-based real-time data acquisition using open-source MQTT brokers [26].

Nevertheless, many smart-home sensing papers adopt MQTT without quantifying QoS behavior inside the deployed sensing framework itself. This creates uncertainty about whether sensor events can be delivered within practical alerting constraints.

### 2.5. Smart-City IoT Frameworks, Edge Analytics, and Gateway Prototyping

The gateway layer is central to practical smart-home monitoring because it aggregates data, provides local analytics, and mediates between sensor nodes and dashboards. Serepas et al. demonstrated a lightweight ESP32-based IoT gateway for smart homes using MQTT/REST, on-site data storage, web-based interaction, and reduced cloud dependence [33]. Rapid prototyping studies also show that open-source hardware, Arduino-style development, Python services, and developer-oriented frameworks can shorten the path from sensor integration to deployable IoT prototypes [34,35]. Recent multi-functional smart-home sensing systems further illustrate the feasibility of low-cost multi-sensor communication in home environments [36,37].

These works support the design philosophy of the present study: local processing should be prioritized for privacy, latency, and reproducibility, while optional cloud synchronization can be treated as a future extension rather than a required dependency.

Smart-city IoT framework studies broaden this perspective by showing how smart homes, smart buildings, smart grids, transport systems, and environmental monitoring can operate as interconnected sensing and service domains [38,39]. Within such frameworks, building-level nodes do not need to solve the entire smart-city problem; instead, they contribute localized sensing, communication, and analytics capabilities that can later be connected to larger city-scale services.

Edge computing and analytics literature further supports the use of a Raspberry Pi gateway as a local decision-support node. Hossain et al. proposed an edge-computing framework for situation awareness in IoT-based smart cities, emphasizing processing close to sensors to reduce latency and support timely decision-making [40]. Cesario similarly highlights the role of analytics in extracting useful patterns from urban data for smart-city applications and services [41]. In the present study, the Raspberry Pi gateway plays this edge role at a small building-level scale by collecting sensor data, storing local CSV logs, supporting dashboard visualization, and performing lightweight statistical interpretation before any future cloud integration.

### 2.6. Synthesis and Research Gap

The reviewed literature converges on five points. First, CO_2_ and multi-parameter IAQ monitoring provide useful indicators of ventilation adequacy but are often deployed as standalone environmental systems. Second, privacy-conscious occupancy and smart-building studies show the value of non-imaging sensing, but they rarely combine IAQ, vibration, and communication-layer evaluation in one framework. Third, vibration-monitoring literature demonstrates the value of triaxial accelerometers for disturbance and condition awareness, but this is usually treated separately from domestic environmental monitoring. Fourth, MQTT studies establish that QoS selection affects latency and reliability, yet many smart-home prototypes adopt MQTT without testing QoS behavior in the actual deployed system. Fifth, smart-city IoT and edge-analytics frameworks emphasize distributed sensing, local processing, and scalable services, but there remains a need for small, reproducible building-level design that connects these concepts to measured environmental, vibration, and communication data. Table 1 summarizes how the present work addresses these gaps simultaneously.

## 3. System Design and Overview

### 3.1. Research Design Overview

This study adopted a design science research (DSR) approach to develop and evaluate an integrated IoT-based home environment and safety monitoring artifact [42]. In DSR terms, the artifact is the combined hardware–software monitoring framework, including the sensing nodes, MQTT communication pipeline, Raspberry Pi edge gateway, dashboard, logging process, and analysis workflow. The methodological goal was not to test a social theory, but to design, build, demonstrate, and evaluate a practical artifact that addresses an identified monitoring gap.

The development process was divided into two implementation phases. Phase 1 focuses on artifact construction and feasibility validation through a locally connected design using serial communication and direct display logic. Phase 2 demonstrates the artifact in a more realistic distributed configuration by separating environmental and vibration sensing into ESP32-S3 nodes and routing data through MQTT to the Raspberry Pi gateway.

The overall evaluation consisted of three linked components. First, a documented weighted design-scoring process was used to justify the chosen sensing configuration. Second, the sensing subsystem was evaluated using controlled IAQ and vibration experiments. Third, the communication subsystem was evaluated using 1000-message MQTT QoS batches under localhost and Wi-Fi settings. This combination aligns artifact design with empirical evaluation, which is consistent with the DSR focus on purposeful artifact creation and evidence-based utility assessment.

### 3.2. Overall System Architecture

The final system adopted distributed architecture with three MQTT sensing publishers and one edge gateway. The indoor environmental node monitored carbon dioxide, temperature, humidity, pressure, and gas-resistance/VOC-trend variables using the SCD41 and BME680 sensors. The vibration node captured triaxial acceleration using the MPU6500-class IMU/9250. The outdoor reference node measured ambient CO_2_ for contextual comparison during integrated validation. All three sensing nodes transmitted measurements to a Raspberry Pi gateway over MQTT on a local wireless network. The gateway hosted the Mosquitto broker, subscribed to the sensor topics, displayed live measurements through a Python Tkinter dashboard, computed the indoor–outdoor CO_2_ difference, and stored synchronized data locally for analysis.

**Figure 1:**
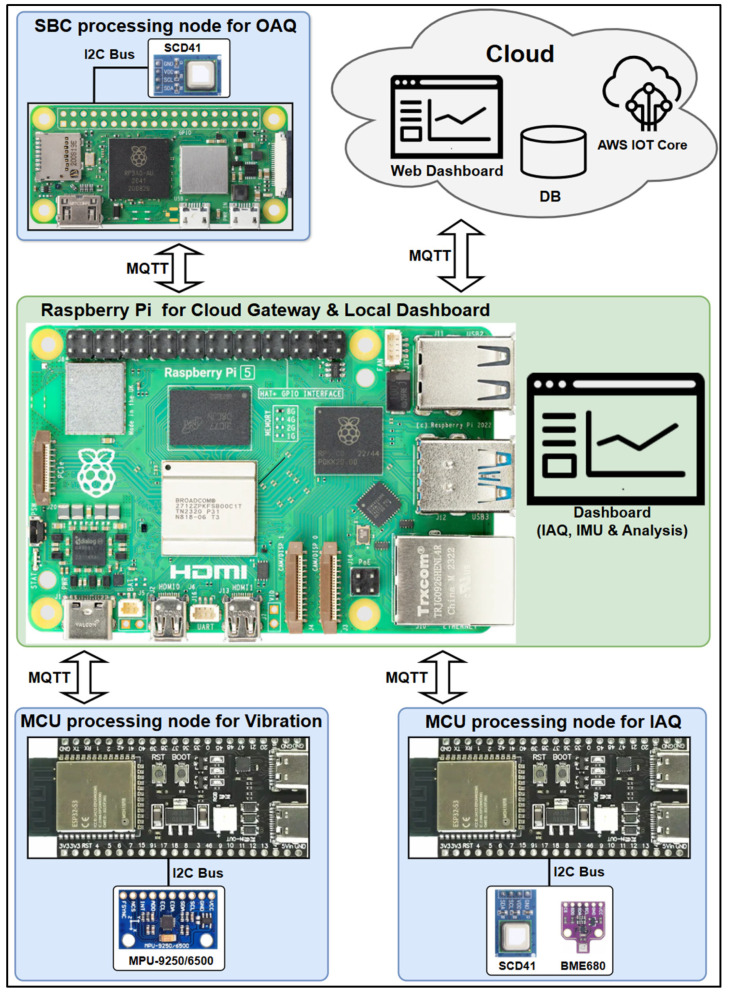
Comprehensive IoT system architecture integrating three MQTT sensing publishers—indoor environmental sensing, local-disturbance/vibration sensing, and an outdoor CO_2_ ambient reference stream—with Raspberry Pi 5 edge processing, MQTT communication, local CSV storage, dashboard visualization, and optional future cloud/mobile extension.

The complete end-to-end architecture of the proposed monitoring framework is illustrated in Figure 1, which shows the interaction between the sensing nodes, MQTT broker, gateway, dashboard, and local storage pipeline.

**Figure 2:**
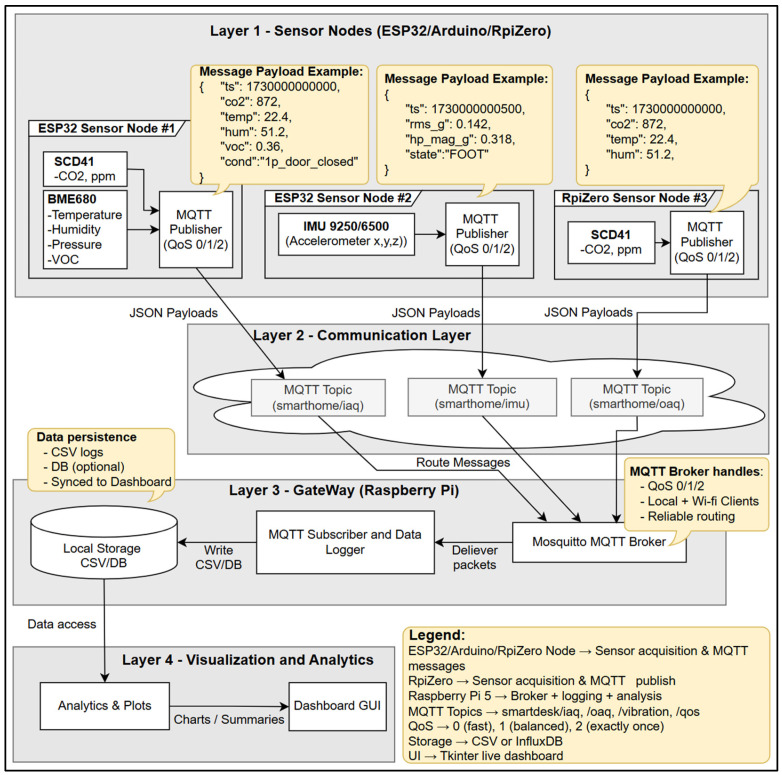
Layered architecture of the proposed framework, showing the sensing layer with indoor IAQ, IMU/vibration, and outdoor CO_2_ reference; the MQTT communication layer; the Raspberry Pi edge-processing layer; the local visualization/storage layer; and the future extensibility layer for mobile or cloud integration.

At a functional level, the system consisted of four coordinated layers: a sensing layer, a communication layer, an edge-processing layer, and an extensibility layer for future cloud or mobile integration. This layered design improved modularity and enabled the system to be extended without changing the core sensing and messaging logic. The layered interpretation of the framework is shown in Figure 2.

### 3.3. Development Phase 1: Feasibility-Stage Design

The first development phase was designed to verify hardware compatibility, signal acquisition, real-time display behavior, and data-logging logic before transitioning to a distributed architecture. In this stage, an Arduino Mega 2560 was connected to the SCD41, BME680, and MPU6500-class IMU sensors. Sensor readings were displayed locally on a 2.8-inch TFT LCD and also transmitted through USB serial communication to a desktop interface developed in .NET WPF.

**Figure 3:**
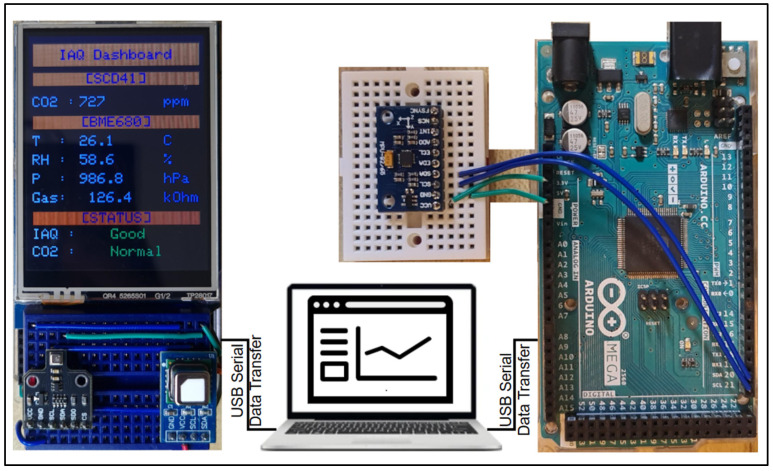
Feasibility stage using Arduino Mega 2560 with SCD41, BME680, and MPU6500-class IMU sensors interfaced to a TFT LCD via USB serial communication.

This feasibility-stage design was not intended as the final system. Instead, it acted as a controlled pre-integration platform in which component behavior could be validated before adding network complexity. This stage was methodologically useful because it helped correct wiring logic, data formatting, and interface behavior early in the project. The feasibility-stage configuration is presented in Figure 3.

### 3.4. Development Phase 2: Final Distributed Design

After feasibility validation, the system was migrated to a distributed edge-IoT architecture comprising two ESP32-S3 DevKitC-1 indoor sensor nodes, an ambient reference publisher, and a Raspberry Pi 5 gateway. The first ESP32-S3 node published indoor-air-quality data from the SCD41 and BME680, while the second ESP32-S3 node published vibration data from the MPU6500-class IMU/9250. The ambient reference publisher, implemented on a Raspberry Pi Zero 2 W, published outdoor CO readings to a separate MQTT topic to support contextual comparison during integrated testing. All sensor streams were transmitted as JSON-formatted MQTT payloads to the Raspberry Pi 5 gateway, which hosted the Mosquitto broker, dashboard processing, edge-side logging, synchronized CSV storage, and local data management. The same Raspberry Pi Zero 2 W was also used in the QoS experiments to emulate a secondary Wi-Fi-connected publisher.

**Figure 4:**
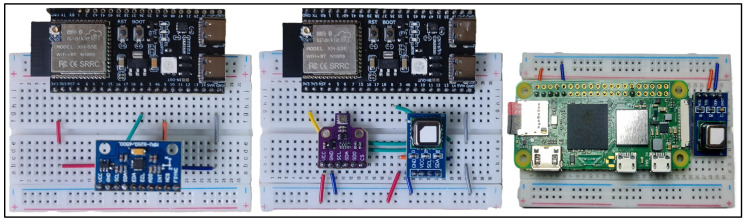
Final distributed configuration showing three MQTT sensing publishers: an ESP32-S3 indoor IAQ node, an ESP32-S3 vibration/IMU node, and a Raspberry Pi Zero outdoor ambient CO_2_ reference node, all publishing to a Raspberry Pi gateway for local logging, dashboard visualization, MQTT health monitoring, and edge-side fusion analysis.

Compared with the feasibility-stage, the final three-node distributed system provided greater modularity, improved extensibility, and a more realistic smart-home deployment model. The final distributed design is shown in Figure 4.

### 3.5. Hardware and Software Configuration

The hardware and software stack was selected to balance cost, reproducibility, sensing capability, and ease of implementation. The SCD41 was selected for direct NDIR carbon dioxide sensing, the BME680 for temperature, humidity, and gas-resistance/VOC-trend monitoring, and the MPU6500-class IMU for vibration-state monitoring. The distributed sensing layer used ESP32-S3 boards for the indoor IAQ and vibration nodes, while an ambient reference publisher provided supporting outdoor environmental measurements for integrated validation. A Raspberry Pi 5 served as the main gateway, providing MQTT broker hosting, local analytics, dashboard visualization, synchronized CSV logging, and local storage. The complete hardware and software configuration used in the study is summarized in Table 2.

The selected hardware was intentionally based on widely available low-cost platforms rather than the newest microcontrollers or sensors. This choice supports reproducibility, affordability, and accessibility for proof-of-concept smart-home and small-building deployments. Future implementations can compare newer microcontrollers, gateway boards, and sensing modules to improve latency, power efficiency, and scalability.

### 3.6. Sampling Logic and Timing Selection

Sampling rates were chosen according to the physical behavior of the monitored variables. Environmental variables such as carbon dioxide, temperature, and humidity change comparatively slowly, so the environmental subsystem was sampled at approximately 0.33 Hz (about one sample every 3 s), with controlled ventilation segments of different durations. By contrast, vibration states change rapidly and therefore required higher temporal resolution. The vibration subsystem was sampled at approximately 100 Hz (about one sample every 10 ms), with short, labelled segments recorded for each controlled disturbance condition. MQTT performance was evaluated separately through batches of 1000 messages per QoS level.

The above-mentioned configuration balanced signal representativeness, storage efficiency, and implementation simplicity. Slower environmental sampling reduced redundant logging while remaining sufficient to capture ventilation-related changes. Faster vibration sampling provided adequate temporal resolution for RMS-based state differentiation. The ambient reference stream was logged at the same gateway level to support contextual interpretation during integrated validation.

### 3.7. Experimental Procedures

Three connected experimental procedures were used. The first focused on sensor relevance and selection through a weighted multi-criteria design-scoring assessment. The second examined sensing behavior through indoor-air-quality and vibration experiments. The third evaluated communication performance through MQTT latency, observed message reception, throughput, and duplicate-message detection under QoS 0, QoS 1, and QoS 2 in both localhost and Wi-Fi settings.

The indoor-air-quality experiment was performed in a compact single-zone room measuring approximately 5 m × 3 m, with a ceiling height of 2.4 m. This corresponds to a floor area of approximately 15 m^2^ and an estimated room volume of 36 m^3^. The room size is consistent with a small-to-medium bedroom or study-room scale in a residential context, making it suitable for controlled proof-of-concept validation. Natural ventilation was controlled through one door and one top-hung awning window, with no mechanical ventilation used. The door measured approximately 1.95 m × 0.75 m, while the window measured approximately 0.25 m × 1.0 m. In the open condition, the door was fully open and the awning window was opened to its maximum available opening. In the partly closed condition, the door was opened to approximately 45° and the window to approximately 30°. In the closed condition, both the door and window were closed.

**Figure 5:**
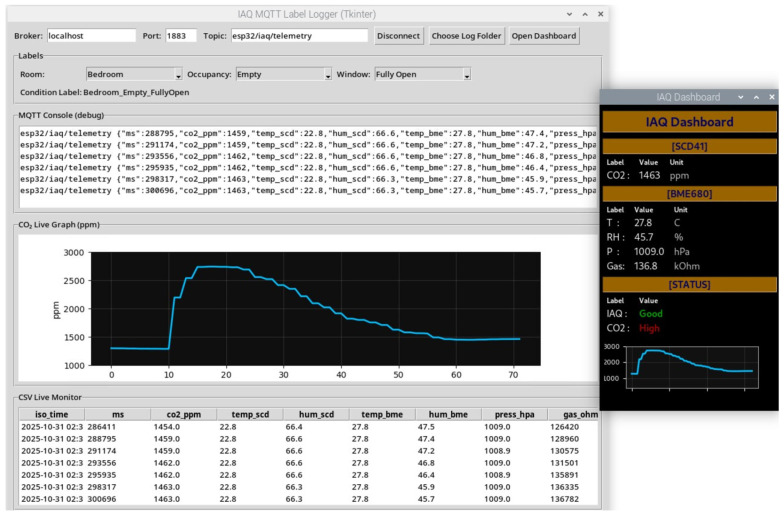
Tkinter dashboard for IAQ monitoring and ventilation-condition labeling, showing real-time CO_2_, temperature, humidity, pressure, and gas-resistance/VOC-trend indicators during controlled room-state experiments.

Although this room does not capture the full diversity of residential and office layouts, it enabled controlled manipulation of occupancy, natural ventilation, and vibration stimuli during prototype validation. The controlled room size and natural-ventilation states allowed CO_2_ changes to be observed clearly within practical experimental windows, while maintaining a realistic small-room context. The experimental conditions, sampling parameters, and durations across IAQ, vibration, and MQTT domains in the study are summarized in Table 3. Figure 5 shows Tkinter dashboard for IAQ monitoring and ventilation-condition labeling during controlled room-state experiments.

### 3.8. Integrated End-to-End Run

The integrated actual run evaluated whether the multi-node MQTT monitoring system could operate as a synchronized edge-IoT pipeline. The run used the indoor IAQ publisher, the IMU/vibration publisher, the ambient reference publisher, and the Raspberry Pi 5 gateway. The gateway subscribed to all relevant MQTT topics, displayed live sensor values in the dashboard, logged synchronized CSV records, monitored message freshness and inter-arrival timing, and assigned gateway-side fusion states.

The run followed five manually selected integrated test segments: T1 baseline empty/door open, T2 occupied/partly closed, T3 disturbance event during the one-person/half-closed-door condition, T4 occupied/door closed, and T5 recovery/door reopened. These segments were used for synchronized logging and integrated analysis, while the term “ventilation condition” was retained when interpreting room-state behavior in relation to the earlier IAQ experiment. The ambient reference stream provided supporting background context for interpreting indoor accumulation during restricted ventilation.

### 3.9. Calibration and Quality Assurance

Calibration and quality assurance were incorporated into both development phases. For the environmental subsystem, the carbon dioxide sensor zeroed against outdoor baseline air at approximately 420 ppm before formal experimentation. For the vibration subsystem, IMU zero-bias calibration was carried out so that the static acceleration magnitude approximated gravitational acceleration under stationary conditions. Repeated drift checks indicated that sensor deviation remained below 2% during the study period.

Quality assurance also included message-format verification, timestamp consistency checks, and validation of CSV logging completeness. In Phase 1, live serial display was used to inspect data continuity. In Phase 2, MQTT topic routing, JSON payload integrity, and gateway-side subscription behavior were checked before full data collection.

### 3.10. Data Processing and Analysis Workflow

All measurements were stored locally in CSV format and processed using Python scripts. Environmental and vibration datasets were cleaned and grouped by experimental condition. Descriptive statistics were then used to summarize distributions. For the air-quality and vibration experiments, one-way ANOVA was applied to test whether condition-dependent differences were statistically significant. Because the measurements were collected as controlled time-series samples, the resulting *p*-values are interpreted as condition-separation evidence rather than population-level generalization. Tukey HSD post hoc comparisons were used where appropriate to identify pairwise separation. For vibration analysis, RMS acceleration served as the primary discriminative feature. For MQTT evaluation, message-level logs were used to compute mean latency, latency standard deviation, observed message-reception rate, throughput, and duplicate count. Because the MQTT latency distributions were non-normal and showed unequal variance, non-parametric Kruskal–Wallis tests were used for QoS-level latency comparisons.

For the integrated end-to-end run, the gateway synchronized indoor environmental data, vibration data, ambient reference measurements, MQTT health indicators, and gateway-side fusion states within a single edge-processing workflow. The fusion logic prioritized message freshness, alert thresholds, rising environmental trends, RMS-based disturbance events, and baseline-band interpretation. The ambient reference stream provided supporting context for interpreting indoor accumulation during restricted ventilation.

### 3.11. Validation Strategy and Methodological Scope

Validation in this study was addressed at three levels. First, design validation was handled through a documented weighted design-scoring process to show that sensor selection was not arbitrary. Second, sensing validation was addressed through statistically significant differentiation of environmental and vibration conditions. Third, communication validation was addressed through repeatable QoS experiments that revealed interpretable latency, throughput, and protocol-level delivery-assurance trade-offs.

The study was intentionally scoped as a proof-of-concept in a compact indoor setting using privacy-conscious sensors, local wireless networking, and lightweight analytics. It did not attempt predictive control, multi-room airflow modeling, cloud-scale deployment, or machine-learning-based anomaly detection. These boundaries helped maintain clarity while establishing a strong experimental baseline.

With the design and implementation established, the next section presents the experimental results.

## 4. Results

### 4.1. Sensor Relevance and Weighted Design-Scoring Results

To make the sensor-selection rationale auditable, the weighting structure was informed by multi-criteria decision-making logic associated with AHP, but this study reports a transparent weighted design-scoring calculation rather than a full pairwise-comparison AHP model [43]. The scores are not direct sensor-output values. Instead, each candidate sensing modality was rated on a 1–5 design-context suitability scale using official manufacturer documentation, peer-reviewed literature, system integration experience, and observed suitability for the target monitoring task. Manufacturer documentation was used for measurable technical characteristics such as sensing modality, measurement range, interface support, response behavior, operating voltage, and power characteristics [44,45,46,47,48]. Peer-reviewed studies were used to contextualize the strengths and limitations of CO_2_ sensing, MOX gas-resistance sensing, vibration monitoring, and occupancy-sensing alternatives [6,11,15,30].

Using this documented scoring approach, the MPU6500-class IMU achieved the highest weighted score because triaxial acceleration directly captured fast local-disturbance events with low computational overhead. The SCD41/SCD40-class CO_2_ sensor scored strongly for sensitivity and reliability because it directly measured ventilation-related CO_2_ concentration, although its environmental response dynamics are slower than inertial sensing. The BME680 provided useful auxiliary temperature, humidity, pressure, and gas-resistance/VOC-trend information, but its reliability score was lower because low-cost MOX gas sensing is non-specific and affected by cross-sensitivity, drift, and ambient conditions. PIR and ultrasonic sensors were considered as candidate technologies during the sensor-selection stage rather than being physically integrated into the implemented system. While both options offer advantages in response speed, cost, and low-power operation, they were ranked lower for this study because they do not directly capture IAQ parameters or vibration-related disturbance conditions.

**Figure 6:**
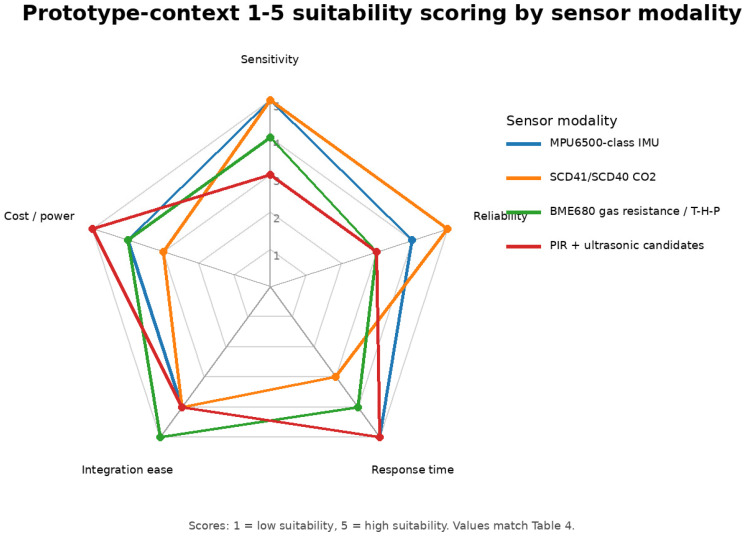
Comparative 1–5 scoring plot for candidate sensor modalities across sensitivity, reliability, response time, integration ease, and cost/power suitability. The plot visualizes the documented suitability scores reported in Table 4.

Figure 6 presents the comparative 1–5 suitability scoring plot of candidate sensor modalities. The radar plot is generated from the documented Table 4 values.

### 4.2. Indoor Air-Quality Results

The indoor-air-quality experiment evaluated whether the environmental subsystem could differentiate ventilation states using low-cost carbon dioxide and environmental sensing. Four controlled conditions were tested: empty/door open, one person/door open, one person/door partially closed, and one person/door closed. After excluding one invalid startup row with CO_2_ = 0 ppm, one-way ANOVA confirmed a statistically significant main effect of ventilation condition on carbon dioxide concentration, F(3, 2902) = 2359.88, *p* < 0.001, with a large effect size, partial eta-squared = 0.709. Tukey HSD post hoc testing showed that all pairwise condition comparisons were statistically significant.

Mean carbon dioxide concentration increased from 395.47 ppm in the valid empty/door-open baseline to 569.86 ppm in the one-person/door-open condition, 680.13 ppm in the one-person/partially closed condition, and 1083.16 ppm in the one-person/closed condition. Based on the firmware classification logic, CO_2_ was interpreted as Normal below 800 ppm, Elevated from 800 to 1199 ppm, and High at 1200 ppm or above. Therefore, the mean CO_2_ level remained within the Normal range for the first three conditions, while the closed occupied condition shifted into the Elevated range. This indicates that restricted ventilation and occupancy produced a clear and measurable deterioration in ventilation-related air quality.

Relative humidity increased by approximately 3.10 percentage points as ventilation became more restricted, while temperature rose by approximately 3.29 deg C across the tested conditions. These changes were smaller than the CO_2_ increase, suggesting that CO_2_ was the most sensitive indicator of ventilation restriction in this experiment. Humidity remained within the commonly used 30–60% comfort reference range, whereas CO_2_ showed a stronger condition-dependent response.

**Figure 7:**
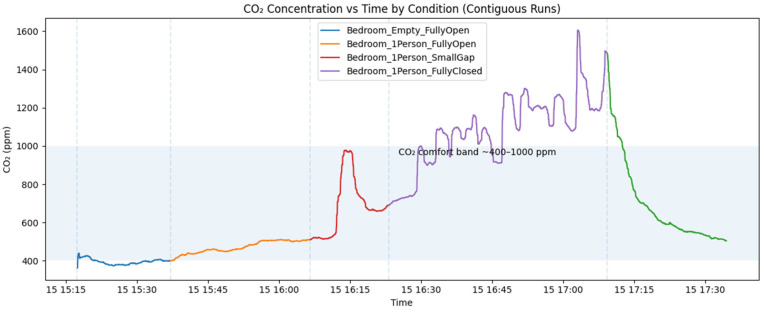
Carbon dioxide concentration across four ventilation conditions, showing a clear increase from the valid empty/open-door baseline to the closed occupied condition.

**Figure 8:**
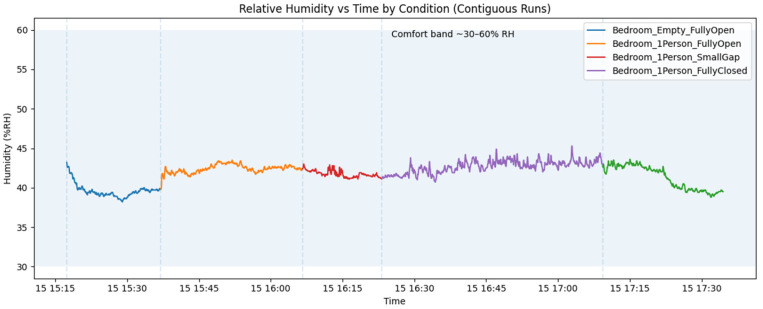
Relative humidity across four ventilation conditions. The shaded reference region indicates the commonly used 30–60% RH comfort range; all tested conditions remained within approximately 39–44% RH while CO_2_ changed more strongly across ventilation states.

A summary of carbon dioxide levels by condition is given in Table 5. The carbon dioxide trend across ventilation states is shown in Figure 7, while the corresponding humidity pattern is shown in Figure 8.

To make the firmware-level interpretation explicit, Table 6 summarizes the CO_2_ status distribution for each ventilation condition using the embedded classification thresholds. The status distribution shows that ventilation restriction changed not only the mean CO_2_ concentration but also the proportion of readings classified as Elevated or High. In the closed occupied condition, 87.1% of valid readings were classified as Elevated or High, compared with 0% in the empty/open-door baseline.

The BME680 gas-resistance output, as shown in Table 7, was also interpreted in the firmware, but it should not be described as calibrated VOC concentration. Instead, it is reported as a gas-resistance/VOC-trend indicator. In the embedded logic, gas-resistance status was classified as Good when gas resistance was at least 80 kOhm, Moderate from 40 kOhm to 79.999 kOhm, and Poor below 40 kOhm. These labels provide a local trend/status flag for the prototype rather than a certified IAQ or VOC measurement.

Overall, the indoor-air-quality results support the environmental subsystem as a useful proof-of-concept indicator of ventilation-state change. CO_2_ provided the clearest separation across the four controlled conditions, while humidity and BME680 gas-resistance status supplied supporting environmental context. Because the experiment was conducted in a compact proof-of-concept test space, future validation should repeat the protocol in standard-sized rooms and multi-room environments to assess scalability, sensor placement effects, and longer-term sensor stability.

### 4.3. Vibration Results

The vibration subsystem was evaluated to determine whether a low-cost triaxial MEMS sensor could distinguish different levels of controlled disturbance. The dataset contained 3979 samples, and RMS acceleration was used as the principal discriminative feature. One-way ANOVA revealed a highly significant effect of vibration class, with F(4, 3974) = 22,486.6, *p* < 0.001. RMS values increased monotonically from the least active class to the highest-intensity disturbance state.

The threshold-based RMS rule produced complete agreement with the labelled states in this controlled dataset, achieving precision = 1.00, recall = 1.00, and F1-score = 1.00. This result should be interpreted as evidence of strong separability under predefined experimental conditions, rather than as independent open-world classifier validation.

**Figure 9:**
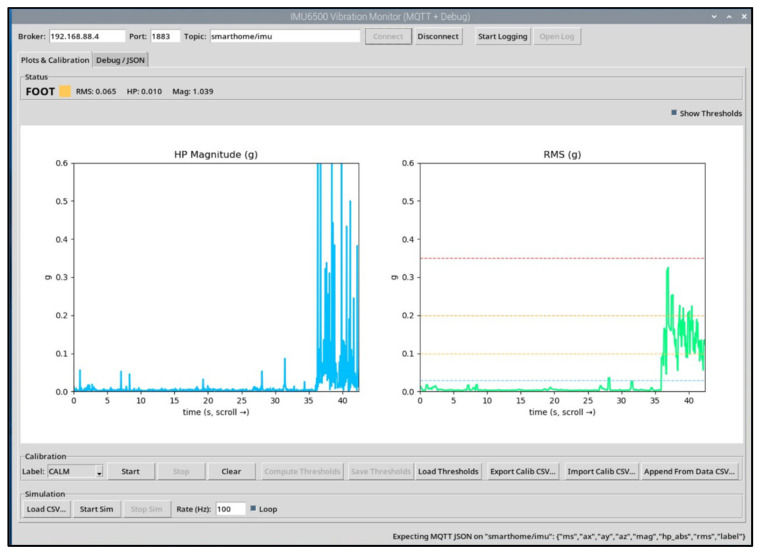
Tkinter dashboard for vibration signal monitoring and state labeling, showing real-time triaxial acceleration streams and assigned disturbance classes for calm, surface-disturbance, footstep, play, and jump conditions.

**Figure 10:**
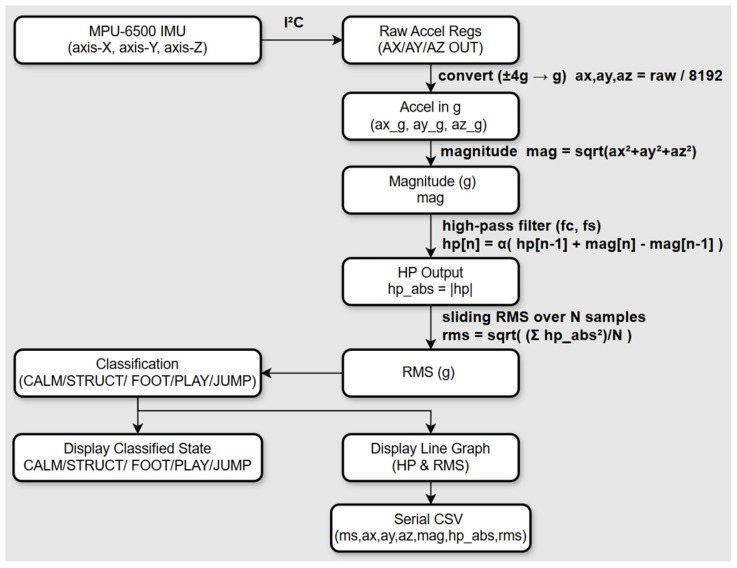
Data-flow diagram for MPU6500-class IMU acquisition, real-time display, graphing, disturbance labeling, and serial CSV logging during the feasibility-stage.

**Figure 11:**
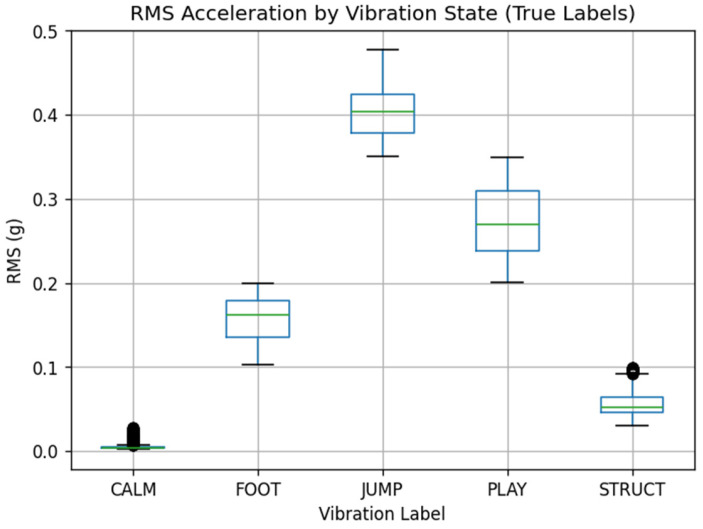
Boxplot of RMS acceleration by vibration class, illustrating separable distribution bands across calm, surface-disturbance, footstep, play, and jump conditions.

The RMS statistics by class are summarized in Table 8. The vibration monitoring interface is shown in Figure 9, the associated signal-display and logging workflow is illustrated in Figure 10, and the classwise RMS distribution is presented in Figure 11.

### 4.4. MQTT QoS Results

The MQTT experiments evaluated communication behavior under QoS 0, QoS 1, and QoS 2 in both localhost and Wi-Fi configurations, with 1000 messages transmitted per condition. Across the uploaded test logs, all QoS and network combinations received 1000/1000 messages, and no duplicates were observed. Therefore, the measured trade-off in this dataset was primarily between latency, throughput, and MQTT protocol-level delivery assurance rather than observed packet loss. Under localhost conditions, mean latency was 0.293 ms at QoS 0, 0.351 ms at QoS 1, and 0.517 ms at QoS 2. Under Wi-Fi conditions, mean latency increased to 25.590 ms, 26.123 ms, and 32.463 ms, respectively.

**Figure 12:**
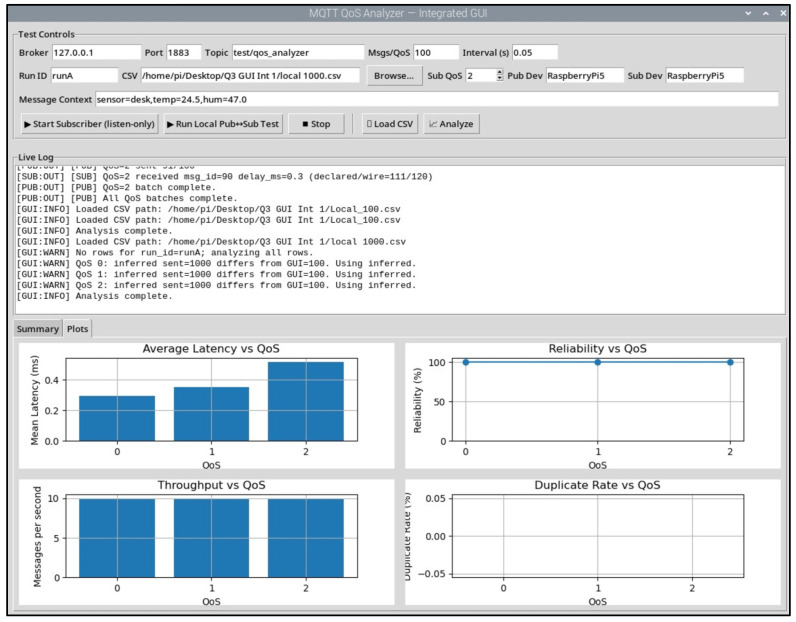
Tkinter MQTT QoS analyzer GUI used to publish and subscribe to 1000-message batches under selected network and QoS configurations while logging latency, throughput, and delivery statistics.

**Figure 13:**
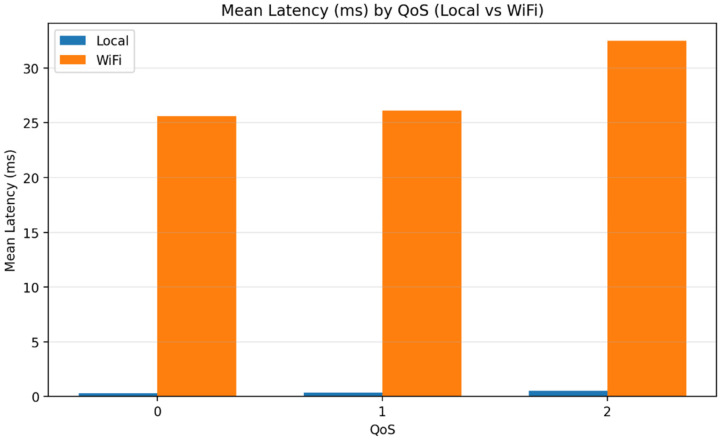
Mean latency versus QoS level under localhost and Wi-Fi configurations, showing the latency overhead associated with higher MQTT delivery-assurance levels.

**Figure 14:**
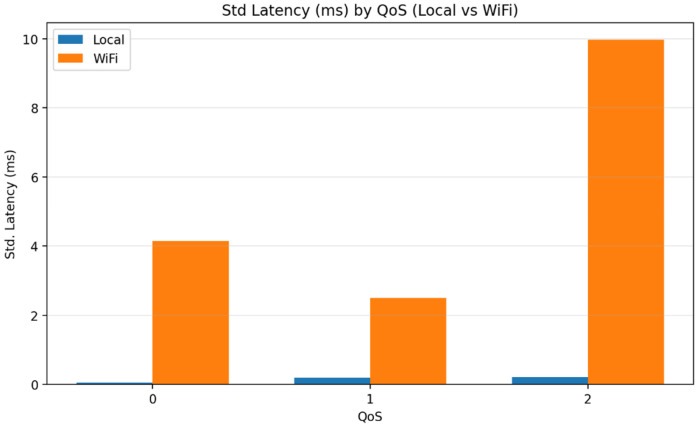
Latency standard deviation across QoS levels, summarizing the variability of message delivery timing under localhost and Wi-Fi configurations.

**Figure 15:**
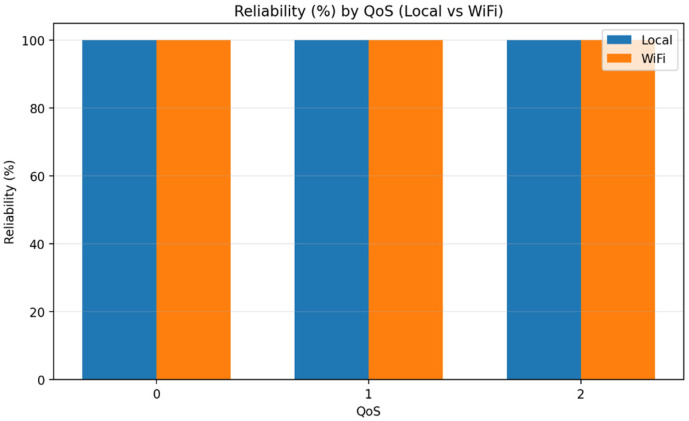
Observed message-reception rate versus QoS level. All tested 1000-message batches achieved complete observed reception with no logged duplicates under the tested localhost and Wi-Fi conditions.

**Figure 16:**
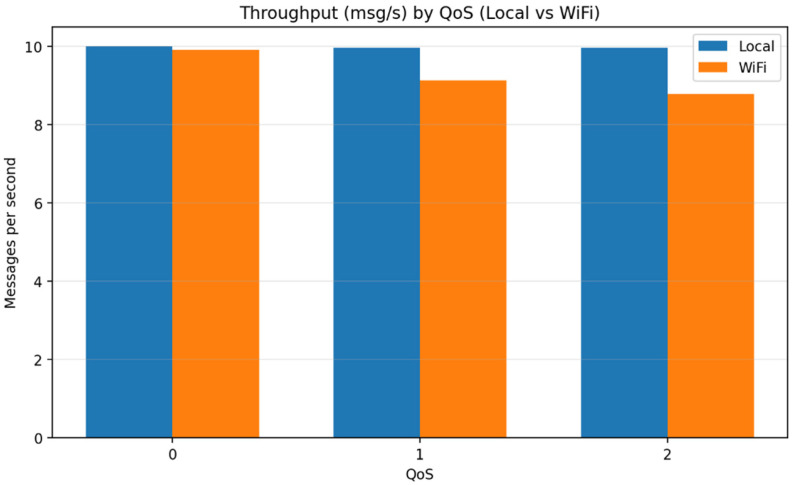
Throughput versus QoS level under localhost and Wi-Fi configurations, showing the trade-off between protocol delivery assurance and message-processing rate.

The overall MQTT QoS performance summary is provided in Table 9. The QoS analyzer interface is shown in Figure 12. The latency trend across QoS levels is presented in Figure 13, latency standard deviation is shown in Figure 14, observed message-reception rate is shown in Figure 15, throughput is shown in Figure 16.

Because the MQTT latency distributions were non-normal and showed unequal variance, Kruskal–Wallis tests were used to compare latency across QoS levels. The tests confirmed significant latency differences for both network configurations: local, H(2) = 1450.28, *p* < 0.001; Wi-Fi, H(2) = 1673.68, *p* < 0.001.

With the empirical results established, the following section interprets their significance in relation to system design, prior work, and practical deployment.

### 4.5. Integrated End-to-End Validation

To address the need for unified system-level evidence, an integrated experiment was conducted in which all sensing components operated simultaneously while measurements were transmitted through the MQTT pipeline to the Raspberry Pi 5 gateway. The indoor environmental publisher reported CO_2_, temperature, humidity, pressure, and BME680 gas-resistance/VOC-trend data from the SCD41 and BME680 sensors. The vibration publisher reported triaxial acceleration from the MPU6500-class IMU. The outdoor reference publisher reported ambient CO_2_ for contextual comparison. The gateway hosted the Mosquitto broker, subscribed to all streams, displayed live values in the Tkinter dashboard, logged synchronized CSV records, monitored MQTT freshness and sequence health, and assigned gateway-side fusion states using transparent rule-based logic.

**Figure 17:**
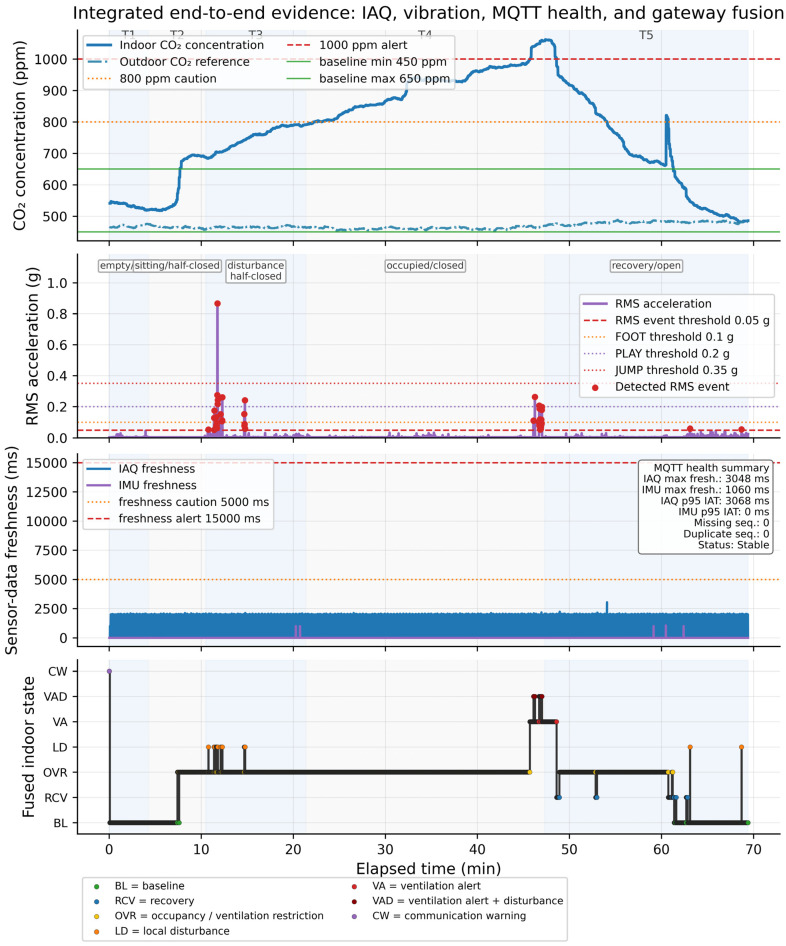
Integrated end-to-end CO_2_ and vibration evidence during integrated end-to-end run. Indoor CO_2_, outdoor reference CO_2_, and RMS acceleration are shown on a common timeline across five manually labeled test segments. Dashed lines indicate the gateway caution (800 ppm) and alert (1000 ppm) CO_2_ thresholds.

The integrated run produced 4107 synchronized gateway records across five manually labelled test segments: T1 baseline empty/door open, T2 occupied/partly closed, T3 disturbance during one-person/half-closed-door condition, T4 occupied/door closed, and T5 recovery/door reopened. During this run, sensing, communication, and edge processing operated concurrently. The synchronized environmental and vibration behavior from this run is summarized in Table 10 and visualized in Figure 17.

Indoor CO_2_ increased progressively as ventilation became more restricted. During T1, the indoor level remained near the baseline band, with a mean of 533.82 ppm, while the outdoor reference remained comparatively stable at 468.49 ppm. In T2 and T3, mean indoor CO_2_ increased to 602.41 ppm and 750.41 ppm, respectively, indicating occupancy and partial-door effects before full door closure. In T4, the indoor trace reached the highest sustained level, with a mean of 910.36 ppm and a maximum of 1060 ppm. This segment crossed the 800 ppm gateway caution threshold and briefly exceeded the 1000 ppm alert threshold, while the outdoor reference remained near 462 ppm. The indoor–outdoor CO_2_ difference increased from 65.32 ppm in T1 to 448.57 ppm in T4, supporting the interpretation that the observed accumulation was associated with the controlled ventilation restriction rather than background outdoor variation alone.

The vibration stream provided complementary local-disturbance evidence. During T3, annotated as the disturbance segment, RMS peaks reached 0.867 g, corresponding to walking, footstep, play, and jump activity while CO_2_ was still below the gateway alert threshold. During T4, RMS remained predominantly low but included short motion bursts under elevated CO_2_ conditions. This allowed the gateway to test combined environmental and disturbance conditions rather than evaluating IAQ and vibration as isolated subsystems. After door reopening in T5, indoor CO_2_ declined gradually, with a segment mean of 705.49 ppm, rather than returning immediately to the T1 baseline. This behavior is expected because indoor air recovery after ventilation restoration is gradual rather than instantaneous.

Humidity and gas-resistance trends provided supporting environmental context. Mean SCD41 relative humidity decreased from 63.49% in T1 to 47.58% in T4, while mean BME680 gas resistance decreased from approximately 334 kΩ to 261 kΩ. These auxiliary variables complemented the CO_2_-based ventilation interpretation but were not treated as selective gas-identification measurements.

Figure 17 shows that indoor CO_2_ increased as ventilation became more restricted, while the outdoor reference remained comparatively stable. The RMS trace provides complementary evidence of local activity, especially during the T3 disturbance segment. Together, these synchronized signals show that the gateway interpreted environmental change and local disturbance within a single integrated MQTT monitoring run.

At the communication layer, all three MQTT publishers remained active throughout the integrated experiment. As summarized in Table 11, sequence-health assessment showed zero missing and zero duplicate flags for the IAQ, IMU, and outdoor reference streams. The IAQ stream produced 1748 unique sequences with a mean freshness of 0.733 s, the IMU stream produced 4102 unique sequences with a mean freshness of 0.001 s, and the outdoor reference stream produced 2052 unique sequences with a mean freshness of 0.518 s. The maximum observed freshness values were 3.048 s for IAQ, 1.060 s for IMU, and 2.054 s for the outdoor reference stream. Therefore, the synchronized traces in Figure 17 were not affected by sequence loss or duplication during the evaluated period, and Table 11 provides communication-layer evidence that the integrated run operated as a stable multi-node MQTT acquisition pipeline.

At the edge-processing layer, the gateway combined environmental, vibration, and MQTT-health evidence using threshold, trend, and freshness rules to assign fused states. During T3, 22 RMS events were logged, producing local-disturbance classifications while CO_2_ remained below the alert threshold. During T4, 18 RMS events occurred under predominantly elevated CO_2_ conditions, allowing ventilation-alert and ventilation-alert-with-disturbance states to be observed when motion coincided with high CO_2_. Overall, the integrated run demonstrates that the proposed system functioned as a unified end-to-end framework in which environmental sensing, vibration sensing, MQTT transport, and gateway-side interpretation operated together in near real time.

Table 10 summarizes the integrated test phases, including phase condition labels, record counts, indoor CO_2_ statistics, indoor–outdoor CO_2_ difference, RMS vibration statistics, RMS event counts, and the primary fused interpretation for each segment.

Table 11 shows that no missing or duplicate sequence flags were observed for the IAQ, IMU, or outdoor-reference streams. Mean freshness remained below 1 s for all streams, indicating that the gateway used recently received MQTT data for synchronized fusion analysis.

## 5. Discussion

### 5.1. Interpretation of the Integrated Findings

The results indicate that the proposed framework functioned as an integrated sensing–communication–analytics pipeline rather than as a set of isolated technical modules. Environmental sensing captured gradual changes associated with ventilation restriction and indoor occupancy, vibration sensing captured short-duration local disturbance events, and MQTT communication provided the transport layer required to deliver these measurements to the gateway in near real time. At the gateway, these streams were synchronized, displayed, logged, and interpreted through rule-based fusion logic. Therefore, the practical value of the system lies not only in its ability to measure indoor conditions, but also in its ability to combine heterogeneous data streams into a single edge-side monitoring workflow.

The integrated end-to-end run confirms this system-level behavior. Indoor environmental measurements, vibration data, MQTT health indicators, gateway-side fusion states, and an ambient reference stream were logged within one synchronized workflow. This shows that the Raspberry Pi gateway operated as more than a passive receiver: it acted as the local coordination point for data acquisition, freshness monitoring, dashboard visualization, synchronized CSV storage, and state interpretation. For practical indoor monitoring, this is important because sensing accuracy alone is insufficient if data are delayed, disconnected, or interpreted separately from communication health.

The combined behavior of the system also demonstrates the value of using sensing modalities with different temporal characteristics. Environmental variables reveal slow and cumulative changes, especially under restricted ventilation, while triaxial acceleration captures abrupt and localized disturbances that are not visible in air-quality data alone. MQTT health monitoring adds a third dimension by showing whether the gateway is receiving recent and complete data from the distributed publishers. Together, these streams allow the framework to observe gradual environmental degradation, sudden dynamic events, and communication status within one monitoring system.

The study also supports the value of a structured design process in low-cost IoT system development. The weighted design-scoring stage justified the selected sensing configuration, while the later controlled experiments and integrated run demonstrated that the selected components produced practically useful outputs under the tested conditions. In this sense, the design-stage scoring and empirical validation reinforced one another. The contribution of the work therefore lies not only in the final distributed artifact, but also in the reproducible pathway used to justify, implement, and evaluate the sensing configuration.

Beyond complementary time scales, contextual interpretation depends on joint CO_2_ and vibration evidence rather than on either stream alone. Rule-based gateway logic maps paired conditions to fused states. High CO_2_ with low vibration—as in much of T4 (mean 910.36 ppm with predominantly calm RMS)—indicates restricted ventilation with limited activity (ventilation_alert). High CO_2_ with high vibration—e.g., brief T4 bursts up to 0.264 g—indicates ventilation stress with concurrent local activity (ventilation_alert_with_disturbance). Moderate CO_2_ with high vibration in T3 (mean 750.41 ppm, 22 RMS events, peak 0.867 g) indicates disturbance under partial ventilation before the closed-door CO_2_ peak. Low CO_2_ with isolated vibration in T1/T5 supports local_disturbance without a ventilation-alert context. Declining CO_2_ after reopening in T5 indicates recovery rather than a new disturbance. As illustrated in Figure 17, fusion is therefore conditional interpretation: each modality constrains the meaning of the other.

### 5.2. Comparison with Prior Work

The findings are consistent with earlier IoT-based indoor monitoring studies showing that environmental sensing can support ventilation awareness in enclosed spaces. However, the present study differs from work that treats indoor air-quality monitoring as a standalone application. Here, environmental sensing was integrated with vibration detection, MQTT QoS evaluation, local dashboard monitoring, synchronized logging, and gateway-side interpretation. This broader integration makes the framework more relevant to practical smart-home and small-building monitoring, where environmental conditions, local disturbances, and communication reliability must be considered together.

The vibration results also align with prior work showing that triaxial accelerometers can support vibration-state and condition-awareness applications. However, rather than focusing on industrial machinery or structural-health monitoring alone, this study positions vibration sensing as part of a domestic safety-awareness pipeline. In this context, the IMU stream does not operate as a separate diagnostic tool; it adds event-level evidence that complements slower environmental sensing. This supports a more complete monitoring model in which gradual environmental changes and sudden local disturbances are interpreted together at the edge gateway.

The MQTT findings further strengthen the system-level interpretation. Previous protocol studies show that MQTT QoS selection affects latency, overhead, and delivery assurance. In this study, QoS behavior was evaluated within the same monitoring framework that handled sensor data and dashboard logging. This is important because communication performance directly affects whether indoor events can be interpreted in time. Under the tested local and Wi-Fi conditions, QoS 1 provided a practical balance between low latency and protocol-level delivery assurance, making it a suitable baseline for similar small-scale edge-IoT monitoring deployments.

### 5.3. Practical Implications

From a practical perspective, the study demonstrates that low-cost open-source hardware can support meaningful indoor environment and safety-awareness monitoring without relying on cameras, microphones, or proprietary cloud infrastructure. The framework is therefore suitable for privacy-sensitive spaces such as bedrooms, dormitories, study rooms, small offices, and assisted-living environments where continuous environmental awareness is useful but intrusive sensing is inappropriate.

The results also show that the gateway layer is central to practical deployment. The Raspberry Pi gateway hosted the broker, subscribed to the distributed topics, displayed live measurements, monitored freshness, logged synchronized records, and supported fusion-state interpretation. This local edge-processing approach improves transparency and allows the system to continue operating without dependence on commercial cloud services. It also makes the system easier to reproduce because the full sensing, communication, and analysis pipeline can be implemented using accessible hardware and open-source tools.

The communication results have direct engineering implications. MQTT QoS should not be treated as a default configuration choice, but selected according to the monitoring requirement. For time-sensitive alerting in the tested local network, QoS 1 offered the most practical balance because it provided delivery assurance with lower overhead than QoS 2. This reinforces the need to evaluate communication settings as part of system design rather than treating networking as a secondary implementation detail.

MQTT and Wi-Fi operate at different layers of the communication stack and should not be treated as direct alternatives. In this study, MQTT was used as the lightweight application-layer publish/subscribe protocol, while Wi-Fi provided the local wireless medium between sensing nodes and the gateway. Therefore, the results evaluate MQTT QoS behavior under localhost and Wi-Fi network conditions, rather than comparing MQTT and Wi-Fi as competing technologies.

The staged development pathway also has practical value. By retaining both the feasibility-stage prototype and the final distributed design, the study presents a realistic progression from component-level validation to networked deployment. This strengthens reproducibility and provides a useful reference for researchers or practitioners who may wish to begin with a simple local prototype before moving toward a distributed sensing architecture.

### 5.4. Scalability and Generalization

The study should be interpreted as a proof-of-concept, building-level implementation rather than a large-scale deployment validation. The 15 m^2^, 36 m^3^ naturally ventilated test room enabled controlled evaluation of sensing response, gateway logging, MQTT communication, and edge-side interpretation. However, it does not represent the full complexity of larger rooms, mechanically ventilated spaces, multi-room homes, office environments, or smart-building deployments. Therefore, the findings provide feasibility evidence for the integrated pipeline, while broader generalization requires further validation.

The MQTT-based architecture provides a practical basis for scaling because sensing publishers communicate through topics rather than fixed wired connections. Additional indoor, outdoor, or room-level reference streams could therefore be added with limited changes to the gateway software. However, larger deployments would require further testing under higher node counts, stronger wireless interference, increased broker load, longer run durations, and more diverse occupancy and disturbance patterns.

Future implementations could also evaluate newer microcontrollers, gateway boards, and sensing modules with improved processing capability, wireless performance, and power efficiency. More recent ESP32 variants, higher-performance Raspberry Pi or edge-computing boards, and alternative environmental sensors may reduce latency, improve buffering, support larger node counts, and extend sensing coverage. However, the main contribution of the present study remains the integrated edge-IoT architecture and reproducible evaluation workflow rather than hardware benchmarking.

The present workflow emphasized descriptive statistics, threshold-based interpretation, and transparent gateway-side logic to maximize reproducibility and implementation simplicity. Future work should validate the framework in standard-sized rooms, multi-room environments, and larger smart-building settings, while also examining calibration drift, sensor placement, network robustness, privacy safeguards, security controls, and adaptive analytics. These steps are necessary before broader deployment can be considered, but the present results establish an initial feasibility baseline for integrated building-level indoor monitoring.

## 6. Conclusions

This study presented a proof-of-concept, building-level IoT artifact for real-time indoor environment and safety-awareness monitoring. Using a design science research approach, the work designed, implemented, and evaluated an integrated hardware–software framework combining indoor environmental sensing, vibration sensing, MQTT communication, local dashboard visualization, CSV logging, and Raspberry Pi edge-side analytics. The system is not presented as a fully deployed smart-city platform; rather, it represents a small-scale building-level implementation that demonstrates how distributed sensing and edge processing could support future smart-home, small-office, and smart-building monitoring applications after further validation.

The experimental results demonstrate the feasibility of the proposed integrated framework. The indoor environmental experiments differentiated controlled ventilation conditions, with CO_2_ increasing from 395.47 ppm in the valid empty/open-door baseline to 1083.16 ppm in the closed occupied condition. The vibration experiments showed separable RMS acceleration bands across calm, surface-disturbance, footstep, play, and jump conditions. The MQTT evaluation showed that all 1000-message batches were received without observed loss or duplication under the tested localhost and Wi-Fi conditions, while latency and throughput varied by network configuration and QoS level. QoS 0 produced the lowest latency, QoS 2 introduced the highest latency overhead, and QoS 1 provided the most practical balance between low latency and protocol-level delivery assurance for time-sensitive alerting in the tested setting.

The integrated end-to-end run further supports the feasibility of the system as a synchronized edge-IoT monitoring pipeline. Indoor environmental data, vibration data, ambient reference measurements, MQTT health indicators, and gateway-side fusion states were logged within one workflow, showing that the framework can combine sensing, communication monitoring, dashboard display, and local analysis rather than operating as isolated modules. Therefore, the main contribution of the study is not a single sensor or algorithm, but the integrated design and evaluation of a low-cost, privacy-conscious, non-imaging monitoring framework for localized indoor safety-awareness assessment.

The study remains limited by its compact 15 m^2^, 36 m^3^ naturally ventilated proof-of-concept test room, short, controlled test windows, and small-scale local network evaluation. These results should therefore be interpreted as feasibility evidence rather than broad deployment validation. Future work should evaluate the framework in standard-sized rooms, multi-room residential or office environments, and larger smart-building settings. Additional work should also compare alternative sensors and wireless protocols, assess long-term robustness and calibration drift, strengthen privacy and security safeguards, and investigate cloud/edge integration strategies. Overall, the findings indicate that lightweight open-source IoT hardware can provide a practical foundation for building-level indoor environment and safety-awareness monitoring, while further large-scale validation is required before broader deployment can be considered.

## Figures and Tables

**Table 1:** Summary of literature gaps addressed by the proposed integrated framework.

Literature Area	Typical Limitation	How This Study Addresses the Gap
IoT-based IAQ and CO_2_ monitoring	Often focused on environmental sensing only	Combines CO_2_, humidity, gas-resistance trend, vibration, and MQTT QoS evidence
Privacy-aware smart-building sensing	Often separates occupancy/environmental sensing from local disturbance detection	Uses non-imaging sensing and integrates environmental and vibration streams
Vibration and condition monitoring	Mostly industrial/structural; rarely connected with home IAQ frameworks	Adapts triaxial RMS acceleration features for indoor disturbance-state differentiation
MQTT communication studies	QoS behavior is often studied separately from deployed smart-home prototypes	Tests 1000-message QoS batches under localhost and Wi-Fi conditions
Smart-city edge analytics	Framework-level studies may not provide small reproducible building-level validation	Implements a Raspberry Pi gateway as a building-level edge node with local logging and analytics
Methodology	Prototype descriptions may lack recognized research framing	Frames the artifact design, demonstration, and evaluation using design science research (DSR)

**Table 2:** Hardware and software components used in the development and evaluation.

Component	Model/Software	Key Specifications	Research Function
Microcontroller Node 1	ESP32-S3 DevKitC-1	Dual-core Xtensa 240 MHz CPU, Wi-Fi/BLE 5.0	Publishes environmental data (CO_2_, temperature, humidity, pressure, and gas-resistance/VOC-trend indication)
IAQ Sensors Node 1	SCD41 (NDIR CO_2_), BME680 (gas resistance + temperature/humidity/pressure)	CO_2_ range 400–5000 ppm ± (40 ppm + 5% of reading)	Indoor-air-quality measurement
Microcontroller Node 2	ESP32-S3 DevKitC-1	Same as above	Publishes vibration data
IMU Sensor Node 2	MPU6500-class IMU (3-axis accelerometer/gyroscope)	±16 g range @ 16-bit resolution	Vibration and structural-activity monitoring
OAQ Sensors Node 3	SCD41 (NDIR CO_2_)	CO_2_ range 400–5000 ppm ± (40 ppm + 5% of reading)	Outdoor-air-quality measurement
Microcontroller Node 3	Raspberry Pi Zero 2 W	Raspbian OS + Python 3.11 + Mosquitto	Publishes ambient data (CO_2_)
Gateway/Server	Raspberry Pi 5 (8 GB RAM)	Raspbian OS + Python 3.11 + Mosquitto	MQTT broker, data logger, GUI visualization
Network Testbed	RPi 5 (localhost) ↔ RPi Zero 2 W (remote)	Wi-Fi 802.11 b/g/n 2.4 GHz	QoS 0–2 latency and reliability evaluation
Initial Stage Testbed	Arduino Mega + TFT 2.8″ LCD	ATmega2560, 4 UART ports	Initial GUI and serial-data validation
Software Stack	Arduino IDE, Visual Studio (WPF), Python (Paho-MQTT, Pandas, Matplotlib)	—	Firmware development and data-analysis environment

**Table 3:** Summary of experimental conditions, sampling parameters, and durations across IAQ, vibration, and MQTT domains.

Domain	Conditions (Labelled)	Sampling Rate	Duration/Run
IAQ	Empty/door open · 1 person/door open · 1 person/door partially closed · 1 person/door closed	≈0.33 Hz (≈3 s interval)	Controlled segments; 2906 valid CO_2_ samples
Vibration	Calm · surface disturbance · footstep · play · jump	≈100 Hz (≈10 ms interval)	≈14–40 s/condition
MQTT QoS	QoS 0, 1, 2 × (Localhost & Wi-Fi)	Batches of 1000 messages	1000 messages/level/ network
Integrated Run	T1 baseline empty/door open · T2 occupied/partly closed · T3 disturbance · T4 occupied/door closed · T5 recovery/door reopened; indoor IAQ + IMU/vibration + outdoor CO_2_ reference	Gateway-synchronized logging	*4107* synchronized gateway records in the final actual run

**Table 4:** Prototype-context weighted design-scoring results for candidate sensor modalities. Scores use a 1–5 suitability scale and are shown in the following order: sensitivity, reliability, response time, integration ease, and cost/power suitability. The technical design rationale combines official manufacturer documentation and peer-reviewed literature.

Sensor Modality	Criterion Scores (Sens.; Rel.; Resp.; Int.; Cost/Power)	Weighted Total	Technical Design Rationale
MPU6500-class IMU/triaxial acceleration	5; 4; 5; 4; 4	4.50	Direct triaxial movement/vibration measurement; official IMU specifications and vibration-monitoring literature support suitability for rapid local-disturbance detection [15,46].
SCD41/SCD40-class CO_2_	5; 5; 3; 4; 3	4.15	Direct CO_2_ measurement for ventilation-state awareness; SCD4x manufacturer specifications and smart-home CO_2_ literature support its IAQ relevance [6,10,44].
BME680 gas resistance/T-H-P	4; 3; 4; 5; 4	3.90	Compact gas-resistance, humidity, pressure, and temperature sensing; reliability score is limited by MOX cross-sensitivity and non-specificity [30,45].
PIR + ultrasonic candidates	3; 3; 5; 4; 5	3.85	Useful for motion/proximity context, but less direct for IAQ and vibration/local-disturbance monitoring; practical use depends on placement and deployment constraints [11,47,48].

Note: Sens. = sensing relevance/sensitivity; Rel. = reliability; Resp. = response time; Int. = integration suitability; Cost/power = combined cost and power-efficiency suitability. The weighted total was calculated as Σ(weight × score), using criterion weights of 0.25 for Sens., 0.25 for Rel., 0.20 for Resp., 0.15 for Int., and 0.15 for Cost/power. Scores represent design-context suitability for this study, not direct laboratory measurements or universal sensor rankings.

**Table 5:** Carbon dioxide concentration by ventilation condition.

Condition	*n*	Mean (ppm)	SD	95% CI Low	95% CI High
Empty/Door Open	421	395.47	14.59	394.07	396.86
1 Person/Door Open	1173	569.86	181.23	559.48	580.24
1 Person/Half-Closed	355	680.13	147.91	664.69	695.57
1 Person/Closed	957	1083.16	190.22	1071.09	1095.22

**Table 6:** CO_2_ status distribution by ventilation condition using firmware thresholds.

Condition	Normal <800 ppm	Elevated 800–1199 ppm	High ≥1200 ppm	Interpretation
Empty/Door Open	421/421 (100.0%)	0/421 (0.0%)	0/421 (0.0%)	Stable normal baseline
1 Person/ Door Open	1062/1173 (90.5%)	97/1173 (8.3%)	14/1173 (1.2%)	Mostly normal with brief peaks
1 Person/ Half-Closed	287/355 (80.8%)	68/355 (19.2%)	0/355 (0.0%)	Normal-to-elevated transition
1 Person/ Closed	124/957 (13.0%)	569/957 (59.5%)	264/957 (27.6%)	Mainly elevated/high

**Table 7:** BME680 gas-resistance status thresholds used for IAQ trend interpretation.

BME680 Gas-Resistance Condition	Firmware IAQ Status	Interpretation
≥80 kOhm	Good	Higher gas resistance; interpreted as a better local gas-resistance/VOC-trend state.
40–79.999 kOhm	Moderate	Intermediate gas-resistance response; interpreted as a moderate local trend state.
<40 kOhm	Poor	Lower gas resistance; interpreted as a poorer local gas-resistance/VOC-trend state.

**Table 8:** RMS acceleration by vibration class.

Label	Count	Mean (g)	SD	Min	Max
CALM	742	0.0055	0.0045	0.002	0.028
STRUCT	905	0.0564	0.0154	0.030	0.100
FOOT	465	0.1577	0.0259	0.102	0.200
PLAY	1285	0.2729	0.0426	0.200	0.350
JUMP	582	0.4019	0.0293	0.350	0.477

**Table 9:** MQTT QoS performance summary under local and Wi-Fi configurations. Observed reception is calculated as received messages divided by 1000 transmitted messages per QoS/network condition.

Network	QoS	Mean Latency (ms)	SD (ms)	Observed Reception (%)	Throughput (msg/s)	Duplicates (Count)
Local	0	0.293	0.059	100	9.993	0
Local	1	0.351	0.194	100	9.955	0
Local	2	0.517	0.216	100	9.961	0
Wi-Fi	0	25.590	4.154	100	9.899	0
Wi-Fi	1	26.123	2.502	100	9.130	0
Wi-Fi	2	32.463	9.970	100	8.774	0

**Table 10:** Summary of integrated end-to-end test segments and integrated test segment-level measurements.

Phase	Condition Label	Records (*n*)	Mean CO_2_ (ppm)	Max CO_2_ (ppm)	Mean Indoor–Outdoor Δ (ppm)	Mean RMS (g)	Max RMS (g)	RMS Events (Count)	Primary Fused Interpretation
T1	Baseline empty/door open	252	533.82	547	65.32	0.0040	0.045	0	Baseline/stable
T2	Occupied/partly closed	368	602.41	695	137.46	0.0031	0.013	0	Occupancy with partial ventilation
T3	Disturbance (half-closed door)	642	750.41	792	284.99	0.0108	0.867	22	Local disturbance under rising CO?
T4	Occupied/door closed	1537	910.36	1060	448.57	0.0051	0.264	18	Ventilation alert ± disturbance
T5	Recovery/door reopened	1308	705.49	1062	225.57	0.0051	0.059	2	Gradual recovery

Note: CO_2_ status in T4 was 96.9% Elevated (800–1199 ppm band). Outdoor reference mean CO_2_ ranged 461.79–479.92 ppm across phases.

**Table 11:** MQTT message-health summary for the integrated end-to-end run.

Stream	Unique Sequences	Missing Flags	Duplicate Flags	Mean Inter-Arrival (s)	p95 Inter-Arrival (s)	Mean Freshness (s)	p95 Freshness (s)	Max Freshness (s)
IAQ (CO_2_, humidity, gas resistance)	1748	0	0	2.33	3.07	0.73	2.05	3.05
IMU (vibration)	4102	0	0	0.0005	0.00	0.001	0.00	1.06
Outdoor CO_2_ reference	2052	0	0	2.02	2.07	0.52	1.06	2.05

## Data Availability

No new data were created or analyzed in this study. Data sharing is not applicable to this article.

## Abbreviations

The following abbreviations are used in this manuscript:

IoT	Internet of Things
IAQ	Indoor Air Quality
OAQ	Outdoor Air Quality
MQTT	Message Queuing Telemetry Transport
QoS	Quality of Service
CO_2_	Carbon Dioxide
VOC	Volatile Organic Compound (used here only as gas-resistance/VOC-trend indication, not calibrated concentration)
MEMS	Micro-Electro-Mechanical Systems
AHP	Analytic Hierarchy Process
RMS	Root Mean Square
NDIR	Non-Dispersive Infrared
